# Analysis of the impact of the combined use of rebar bolts and FRP bolts in the roadway enclosure

**DOI:** 10.1038/s41598-023-46432-1

**Published:** 2023-11-10

**Authors:** Jinguo Lyu, Linfan Qi, Xuebin Wang, Yisheng Peng, Wenhe Han, Shixu Li

**Affiliations:** School of Mechanics and Engineering, Liaoning Technical University, Fuxin, 123000 Liaoning China

**Keywords:** Engineering, Civil engineering

## Abstract

In order to investigate the support effect of the combination of FRP bolts and rebar bolts in the roadway, taking a coal mine as the background of the project, research and analysis of the engineering geological conditions of the mine and the layout of the mining roadway, stress analysis of the roadway peripheral rock, and put forward the combination of rebar and FRP bolts in the roadway peripheral rock support program. Based on different scenarios, FLAC3D was applied to simulate and analyze the distribution of axial force, maximum principal stress of the surrounding rock, yield damage of the surrounding rock, and displacement of the surrounding rock under three conditions: no support, full rebar bolt support, and combined rebar and FRP bolt support. The results show that (1) In the pre-action period between the bolt and the surrounding rock of the roadway, the FRP bolt carries the force first; in the late action period, the rebar bolt and the FRP bolt carry the force together. (2) From the analysis of the stress concentration degree of the maximum principal stress of the roadway surrounding rock, the horizontal displacement of the roadway surrounding rock and the distribution characteristics of the plastic zone of the roadway surrounding rock, it can be concluded that the support strength of FRP bolts is slightly lower than that of rebar bolts. (3) Under the state of combined support of FRP and rebar bolts, the range of plastic zone of surrounding rock in the roadway is analyzed in comparison with the effect of full rebar bolt support, and the range of reduction of plastic zone of surrounding rock is not obvious, and the effect of full rebar bolt support and combined support of FRP and rebar bolts on controlling the damage of surrounding rock is similar. (4) The side part of the roadway perimeter rock mining adopts FRP bolts instead of rebar bolts, and if the FRP bolts are not damaged, the combination of FRP and rebar bolts can be used for support, which can maintain the stability of the roadway perimeter rock.

## Introduction

With the advancement of rod material and support technology, high strength, high elongation, high impact strength bolts and high prestressing support technology have been successfully applied in complex surrounding rock roadways, and all deformation control of the roadway surrounding rock has produced positive results. Today, bolt support has become the primary support form for coal mine roadways in China. With the increase of coal mining depth, the support of roadway has become an important factor to ensure the safety of coal mining^1–6^.

A new kind of corrosion-resistant bolt, FRP bolt^7,8^ has the benefits of being cuttable, having a high tensile strength, being less expensive, being lightweight and strong, having fatigue resistance, having reduced vibration and noise, and more^9–14^. Research and application have been made in the field of coal mine and slope support, and the use of FRP bolts can avoid issues like gas explosions caused by sparks from cutting caused by using metal bolts, damage to the roller cut-off teeth caused by getting caught in the coal miner's drum, and endangering the safety of transportation equipment and personnel by mixing the abandoned bolts into the coal body. A series of pull-out tests were conducted on FRP bolts, and the results demonstrated that FRP bolts can meet boltage requirements under continuous loading conditions. Zhang et al.^15^ first highlighted the great potential of FRP bolts to replace metal bolts under prestressed support conditions in 2004. Ye et al.^7^ explored the support impact of FRP bolts and rebar bolts in 2020 using a combination of field data and numerical calculations. Compared to rebar bolt, the results show that FRP bolt support is relatively less sensitive to roof thickness change, and the support effect is better. By comparing the axial tension values of the two types of bolts, it was shown that FRP bolts may give more axial tension with increased roof thickness, which explains why they provide better bolt support. Numerous studies have shown that employing FRP bolts rather than rebar bolts is a workable solution to the corrosion failure of bolts in metal bolt-supported roadways under specific operational circumstances.

According to a different study, metal bolts also have some benefits for supporting the surrounding rock in the pavement. Li et al.^16^ experimentally investigated the load-carrying capacity of five new rockbolts under axial and shear loadings. The yielding style rockbolts provided considerably more tensile load capacity and deformation compared to the inflatable rockbolts; however, the inflatable rockbolts have the ability to deform significantly more in shear than in tension and have similar shear deformation as the yielding-style rockbolts. Li et al.^17^ experimentally compares the shear behavior of fiber glass (FG) bolt, rock bolt (rebar bolt) and cable bolt for the bolt contribution to bolted concrete surface shear strength, and bolt failure mode. Test results showed that the shear stiffness of FG bolted joints decreased gradually from the beginning to end, while the shear stiffness of joints reinforced by rock bolt and cable bolt decreased bi-linearly, which is clearly consistent with their tensile deformation modulus. The rock bolt contribution to joint shear strength standardised by the bolt tensile strength was the largest, followed by cable bolts, then FG bolts. Through numerical simulation, Guo et al.^8^ compared and examined the deformation and failure characteristics of soft layered roofs supported by rebar bolts and FRP bolts in 2021. The findings indicate that the rebar bolt has a stronger ability to resist the transverse shear force between the rock layers, whereas the FRP anchor has a superior settlement control impact on the weak layered roof. In this situation, the rebar bolt's radial shear resistance is higher in addition to its axial tension^8,18,19^, making the metal bolt with more anchoring techniques more advantageous.

In summary, rebar bolts have the threat of easy corrosion failure in long-term support effect^12^, while FRP bolts have a slight disadvantage in dealing with the lateral shear force of the rock formation. Although the application of combined rebar bolts and FRP bolts in engineering, the systematic analysis of stress, displacement, plastic zone volume and damage type of the tunnel envelope under combined support conditions, as well as the specific axial force distribution characteristics and support process during combined support of both anchors, is not well developed.

In view of this, the author systematically analyzed the combined support effect of two types of bolts, as well as the specific axial force distribution characteristics and support process of the combined support of two types of bolts by numerical simulation software with a typical coal mine as the engineering background, in order to provide reference for the application of FRP bolts in the support of roadway surrounding rock.

## Mine geological profile and support scheme

### Geological conditions

**Figure 1:**
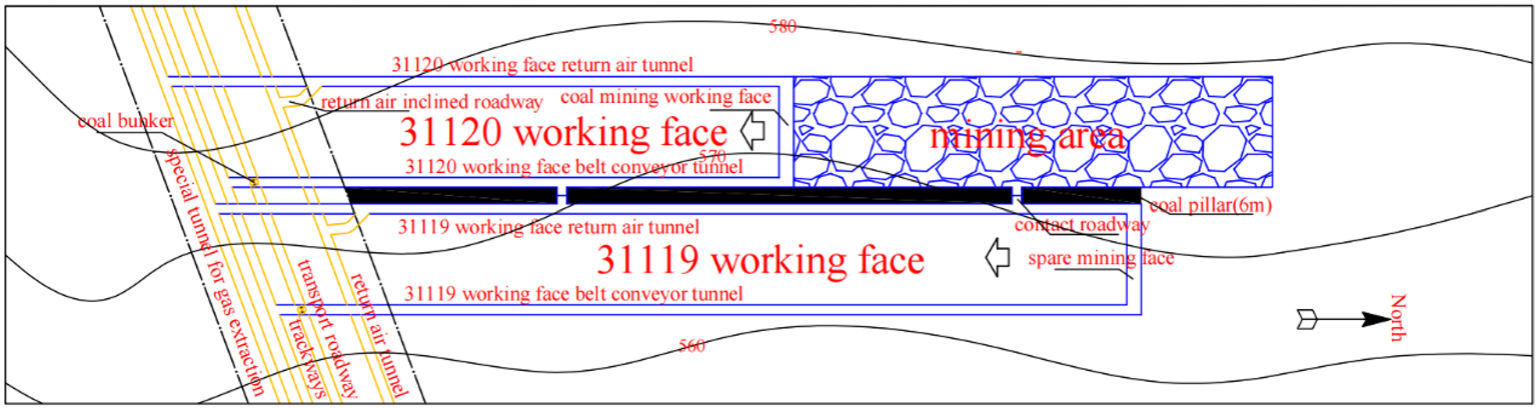
Overview of the work surface.

A mine in Inner Mongolia 31119 faces south of the auxiliary transportation alley, north of the protection of the coal pillar, and west of the 31,120 mining airspace. Working face roadway excavation along the floor, roadway depth of 600 m, the overlying rock layer of self-gravitational stress of approximately 15 MPa, and working face back to the airway along the 6 m small pillar digging. The structure of the coal seam is simple and steady. The coal seam contains 1–2 layers of dirt band, with a thickness of about 0.1–0.2 m. The dirt band lithology is sandy mudstone, dark gray and gray, containing plant fossils and mica debris. The direct roof of the coal seam in the working face is 4.34 m thick sandy mudstone, dark gray, horizontal bedding, containing a large amount of coal debris and plant fossils, with good shale content and flat fracture. The upper main roof is 10.94 m siltstone, light gray, thin layer structure, near horizontal bedding, fracture development, local thin layer fine sandstone, coal line, and plant fossils. The base of the coal seam is sandy mudstone, dark gray, horizontally laminated, containing a large amount of coal debris and plant fossils, with good mud content. The overview of the working face is shown in Fig. 1.

According to the geological column map of this typical coal mine and the description of the support materials, combined with the actual engineering conditions and stratigraphic information on the site, it is possible to determine the range of values of the physical and mechanical parameters of the rock layers and determine the physical and mechanical parameters of the coal and rock layers, see Table 1. These parameters play a crucial role in the reliability of the numerical simulation results.

**Table 1:** Physical and mechanical parameters of coal rock formations.

Rockiness	Density (kg·m^−3^)	Bulk modulus (GPa)	Shear modulus (GPa)	Angle of internal friction (°)	Cohesion (MPa)	Tensile strength (MPa)
Sandy mudstone	2510	2.56	2.36	36.5	2.26	1.21
Coal seam	1314	2.918	0.987	34.3	1.98	1.39
Sandy mudstone	2540	2.56	2.36	36.5	2.26	1.21
Fine-grained sandstone	2510	5.56	3.92	23.25	2.50	1.35

### Roadway bolt (cable bolt ) support scheme

#### Option 1: Full rebar bolt support

**Figure 2:**
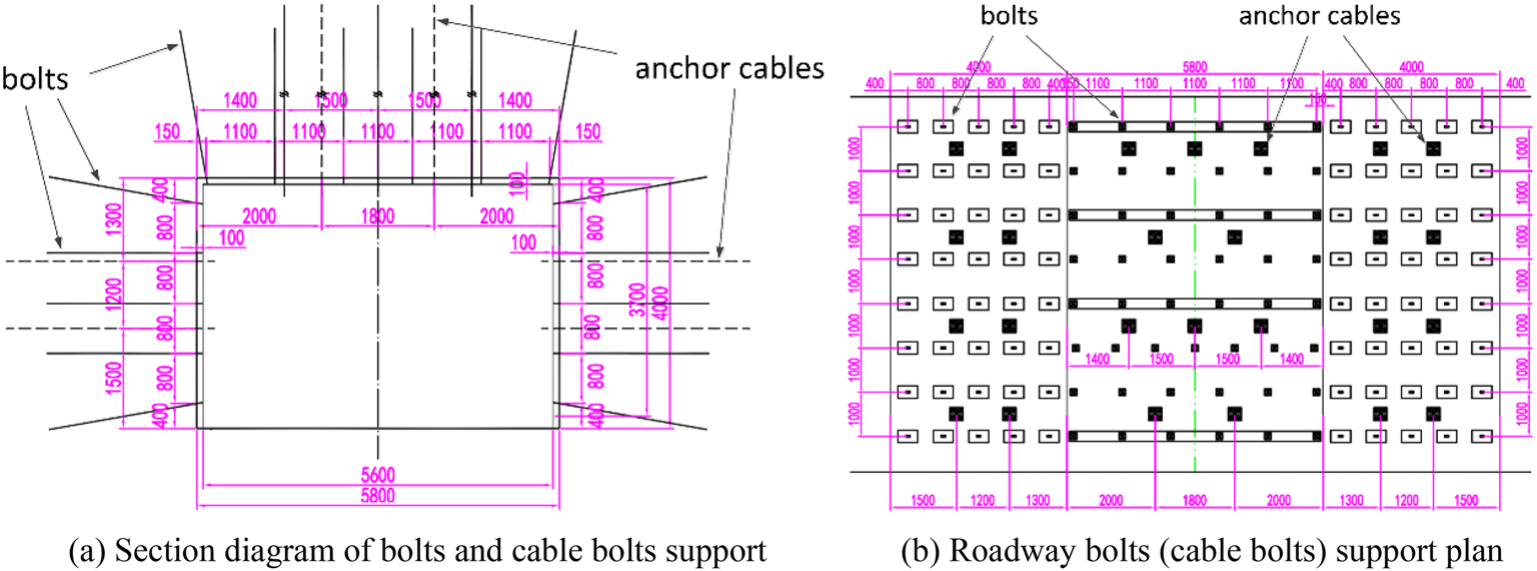
Schematic diagram of the layout of the bolts (cable bolts) support of the mining roadway.

**Figure 3:**
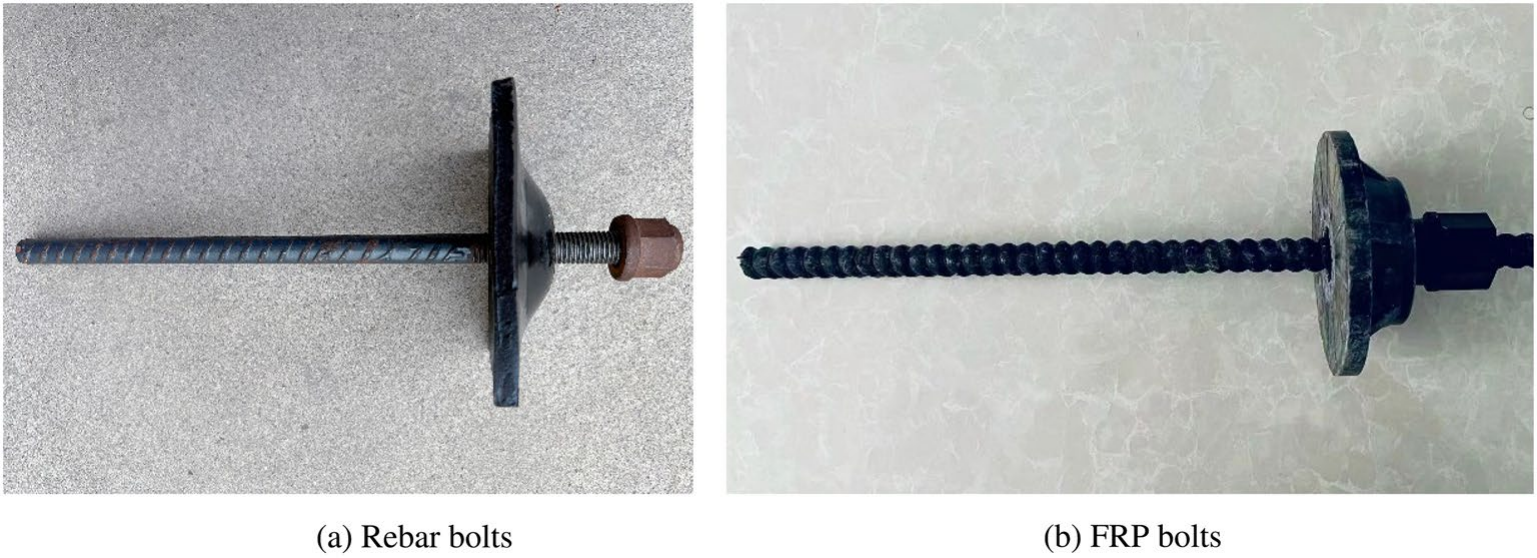
Bolt schematic diagram.

The design of the roadway supportopt is shown in Fig. 2a, the roadway is dug along the bottom of the coal seam, with a rectangular cross-section, a width of 5800 mm, and a height of 4000 mm. The bolts and anchor cables are marked in the illustration. Bolt specifications: Rebar bolts are made of high-strength low alloy steel, see Fig. 3a, the bolt of roadway roof is made of φ22 × 2400 mm rebar bolt, and the yield strength of the bolt is not less than 500 MPa., the thread at the end of the rod is M24, and the thread length is not less than 150 mm, using high strength nut M24 × 3.0, Bolts bolting method: Each bolt is equipped with two CK2350 resin bolts. Specification of anchor cable in the sidewall: φ21.8 × 3150 mm, and the anchor cable material is 1 × 19 strands of high strength low relaxation prestressing steel strand, bolting method of anchor cables in the sidewall: each anchor cable is equipped with three CK2350 resin bolts. Roadway roof cable bolt specification: φ21.8 × 6300 mm, anchor cable material is 1 × 19 strands of high strength low relaxation prestressing steel strand, anchoring method of cable bolt at the roof of roadway: each bolt with 3pcs of CK2350 resin bolting agent.

Arrangement of two sides of roadway bolt: 5 bolts per row, see Fig. 2b for inter-row spacing, and roof bolt arrangement of roadway: 6 bolts per row, see Fig. 2b for inter-row spacing.

#### Option 2: Combined FRP bolt and rebar bolt support

The rebar bolt on the left side of the roadway is replaced by FRP bolt, FRP bolts are made of fiberglass reinforced material, see Fig. 3b, and the support scheme for other locations is the same as Option 1.

## Analysis of roadway peripheral rock stress and bolt (cable bolt) support resistance

### Analysis of the mechanical characteristics of the roadway enclosure

So far, the equivalent circle approach, the complicated function method, and the pressure arch method are the major ways to solve the loosening circle of rectangular roadway. In this study, the loosening circle of the circular roadway is calculated, the loosening circle of the rectangular roadway is established based on the loosening circle of the circular roadway, and the range of the plasticity zone of the rectangular roadway is ultimately determined.

**Figure 4:**
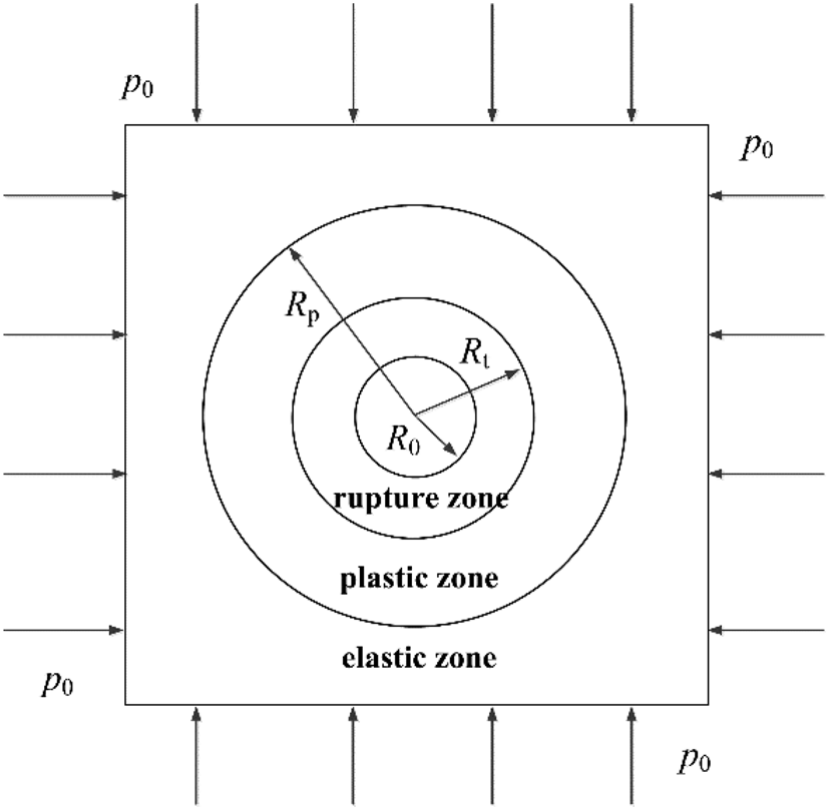
Schematic diagram of the surrounding rock mechanics of the roadway.

A circular tunnel is provided with a radius of ${R}_{0}$ and an original stress of ${p}_{0}$. The surrounding rock consists of three parts: the fracture zone (radius ${R}_{\mathrm{t}}$), the plastic zone (radius ${R}_{\mathrm{p}}$) and the elastic zone (see Fig. 4).

Based on the equivalent circle method, it is known that the tangential stresses σ_θ in the elastic and plastic zones of the circular roadway are distributed as follows according to the Mohr–Coulomb criterion counting and elasticity theory^20^:

1 $$\sigma_{{\uptheta }}^{{\text{e}}} = p_{0} \left( {1 + \frac{{R_{{\text{p}}}^{2} }}{{r^{2} }}} \right) - C{\text{cot}}\varphi \left( {\left( {\frac{{R_{{\text{p}}} }}{{R_{0} }}} \right)^{{\frac{{2{\text{sin}}\varphi }}{{1 - {\text{sin}}\varphi }}}} - 1} \right)\frac{{R_{{\text{p}}}^{2} }}{{r^{2} }}$$

2 $$\sigma_{{\uptheta }}^{{\text{p}}} = C{\text{cot}}\varphi \left( {\frac{{{1} + {\text{sin}}\varphi }}{{1 - {\text{sin}}\varphi }}\left( {\frac{r}{{R_{0} }}} \right)^{{\frac{{2{\text{sin}}\user2{\varphi }}}{{1 - {\text{sin}}\user2{\varphi }}}}} - 1} \right)$$

where: ${\sigma }_{\uptheta }^{e}$ is the tangential stress in the elastic zone; ${\sigma }_{\uptheta }^{p}$ is the tangential stress in the plastic zone; *r* is the distance between any point in the surrounding rock and the center of the equivalent circular roadway; ${p}_{0}$ is the original rock stress, and ${R}_{\mathrm{p}}$ is the radius of the plastic zone; ${R}_{0}$ is the radius of the circular roadway; *C* is the cohesion of the rock; *φ* is the angle of the internal friction of the rock.

Because the elastic and plastic boundaries of the roadway are equal about ${\sigma }_{\uptheta }$, the plastic zone radius is obtained by associating Eqs. (1) and (2):

3 $$R_{{\text{p}}} = R_{0} \left( {\frac{{\left( {1 - \sin \varphi } \right) \times \left( {k\gamma H + C \times ctg\varphi } \right)}}{C \times ctg\varphi }} \right)^{{\frac{1 - \sin \varphi }{{2\sin \varphi }}}}$$

4 $$h_{d} = R_{s} - b/2$$

The minimum support force required to maintain the zone of limiting equilibrium:

5 $$P_{{\text{n}}} = \sum {\gamma h}$$

where: ${R}_{\mathrm{p}}$ is the radius of the plastic zone of the roadway, ${R}_{0}$ is the radius of the outer circle of the roadway, 4 m, *γ* is the average capacity weight of the coal body, taken as 13.6 kN/m^3^, *H* is the burial depth of the roadway, 480 m, *C* is the cohesive force of the surrounding rock, 1.98 MPa, *φ* is the internal friction angle of the surrounding rock, 37.3°, *b* is the height of the roadway, 4.15 m, *h* is the thickness of the roof rock load, ${P}_{\mathrm{n}}$ is the required to maintain the limit equilibrium zone minimum support force required to maintain the limit equilibrium zone.

In order to find the range of the loosening circle of the rectangular roadway, it can be transformed into finding the plastic zone of the circular roadway equivalent to it. Let the width and height of the rectangular roadway be 2*a* and 2*b*, respectively, then the maximum radius of the equivalent circular roadway ${R}_{0}=\sqrt{{a}^{2}+{b}^{2}}$, which can be substituted into the Eq. (3) to obtain the radius of the equivalent circular roadway's loosening circle, therefore, the range of the loose circle of the rectangular roadway roof ${h}_{\mathrm{ct}}$ and the range of the loose circle of the two sides ${h}_{\mathrm{cs}}$ are:

6 $$h{\text{ct = }}R{\text{p}} - b$$

7 $$h{\text{cs = }}R{\text{p}} - a$$

where: *a* is half of the width of the rectangular roadway, 2.5 m; *b* is half of the height of the rectangular roadway, 2.075 m.

Substituting geotechnical parameters into formula (3), ${R}_{\mathrm{p}}$ = 5.88 m, substituting into formula (4), ${h}_{\mathrm{d}}$ = 3.80 m, substituting into formula (5), the minimum support force required to maintain the limit equilibrium zone, ${P}_{\mathrm{n}}$ = 51.68 kN/m^2^, and substituting into formula (6) and (7), the height of the loosening circle of the roof plate of the roadway, ${h}_{\mathrm{ct}}$ = 3.805 m, and the range of loosening circles of both sides ${h}_{\mathrm{cs}}$ = 3.38 m, the value of the loose circle provides the basis for the optimization of the roadway support in the future.

### Bolt (cable bolt) support resistance analysis

#### Resistance analysis of single bolt (cable bolt) support

Distribution of bolt (cable bolt) bond stress along the rod^21^:

8 $$\tau \left( x \right) = \frac{ptx}{{2\pi a}}e^{{ - \frac{1}{2}tx^{2} }}$$

Among them:

9 $$t = \frac{1}{{\left( {1 + \mu } \right)\left( {3 - 2\mu } \right)a^{2} }}\left( {\frac{{E_{0} }}{{E_{b} }}} \right)$$

where: ${E}_{0}$ and *μ* are the elastic modulus and Poisson’s ratio of the geotechnical body, respectively, *a* is the radius of the bolt body, ${E}_{\mathrm{b}}$ is the elastic modulus of the bolt body, *P* is the preload force applied to the bolt body.

Taking the derivative of *x* to its extreme value, it can be obtained that, when $x=\sqrt{1/t}$, the maximum pullout resistance of a single bolt (cable bolt) can be obtained as:

10 $$P_{u} = \frac{2\pi a}{{\sqrt t }}e^{{ - \frac{1}{2}}} \tau_{s}$$

#### Bolt (cable bolt) combination support resistance analysis

1. Maximum support resistance that can be provided by the distribution of multiple anchor cable spacing^22^:
  11 $$P_{s} = 2P_{u} /B \times D$$
  where: ${P}_{\mathrm{u}}$ is the support resistance provided by a single bolt (cable bolt), *B* is the width of the roadway, *D* is the cable bolt spacing.
2. The maximum support resistance that can be provided by the spacing distribution of multiple bolts:
  12 $$P_{m} = \eta \left( {P_{u} /D_{m}^{2} } \right)$$
  where: ${P}_{\mathrm{u}}$ is the support resistance provided by a single bolt (anchor cable), ${D}_{\mathrm{m}}$ is the bolt spacing.
3. The maximum support resistance provided by the combination of bolt and cable bolt support:
  13 $$P = P_{s} + P_{m}$$

By substituting the parameters in the design scheme into Eq. (10), the support resistance provided by a single rebar bolt is 29.92 kN, the support resistance provided by a single FRP bolt is 29.69 kN and the support resistance provided by a single anchor cable is 112.19 kN. By substituting the parameters in the design scheme into Eq. (11), the support force provided by the spacing distribution of multiple anchor cables is 44.88 kN/m^2^. Substituting the parameters in the design scheme into Eq. (12), the support resistance provided by the spacing distribution of multiple rebar bolts is 14.59 kN/m^2^, the support resistance provided by the spaced distribution of multiple FRP bolts is 14.47 kN/m^2^.

The total support resistance provided by the combination of bolt and anchor cable is 59.47 kN/m^2^, and the total support resistance provided by the combination of FRP bolt and anchor cable is 59.35 kN/m^2^, calculated by substituting the above support resistance of bolt and anchor cable into Eq. (13).

In summary, as the total support resistance provided by the combination of rebar bolt and anchor cable support is 59.47 kN/m^2^ greater than the minimum support force of 51.68 kN/m^2^ required to maintain the limit equilibrium zone, the total support resistance provided by the combined FRP bolt and anchor cable support is 59.35 kN/m^2^ greater than the minimum support force of 51.68 kN/m^2^ required to maintain the limit equilibrium zone. Therefore, the support design is safe and serves as a support for the roadway envelope.

## Numerical simulation

### Numerical modeling of the roadway envelope

**Figure 5:**
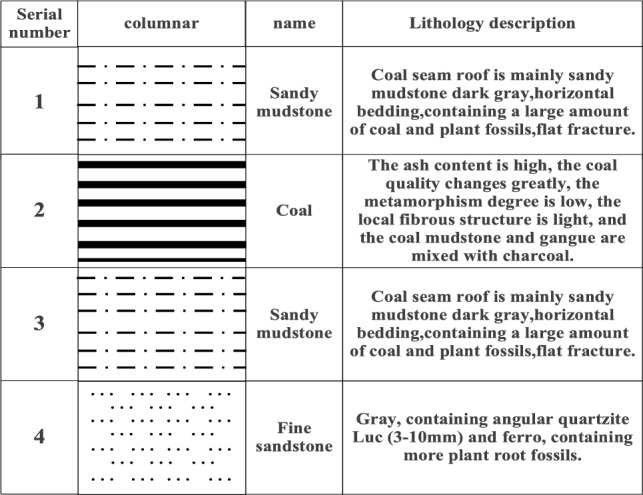
Coal seam histogram.

**Figure 6:**
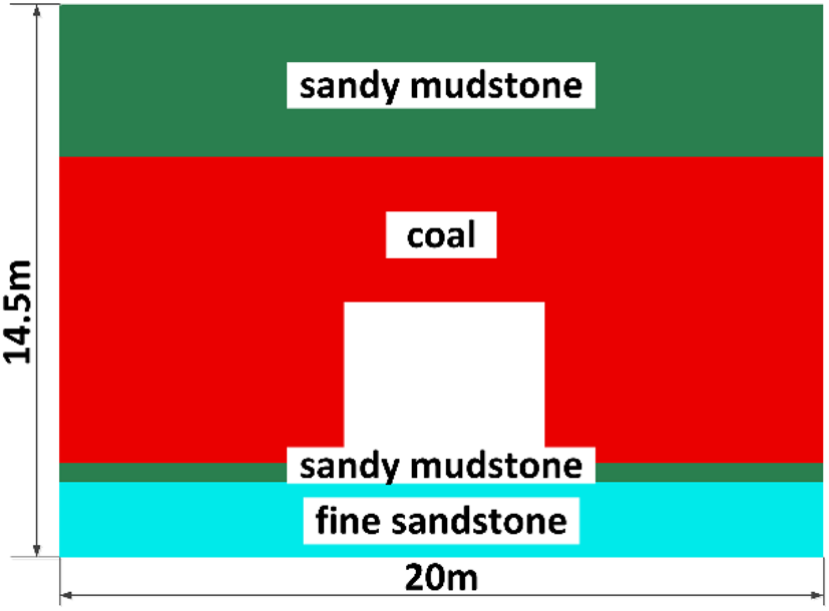
Numerical simulation model diagram.

Three sets of three-dimensional numerical models were constructed for the roadway design scheme in the study region of this typical coal mine using FLAC3D finite element software to numerically simulate the coal seams in the roadway. The length, width and height of the model are 20 m × 10 m × 14.5 m, horizontal displacement constraints are applied to the left, right, front and rear boundaries of the model, and the bottom boundary of the model is fixed. The buried depth of the model is not the real buried depth, and the remaining depth is simulated by applying the equivalent stress on the top. The real burial depth is about 600 m, due to the relatively simple geological structure, the main self-gravity stress, according to the actual experience and mining theory, 15.0 MPa uniform load is applied on the top of the model. The grid cells are densely divided in the tunneling support area, and the grid cells in other areas are relatively sparsely divided. The grid cells are hexahedral cells, and a representative 3D numerical model is selected, as shown in Fig. 6, with reference to the columnar diagram of the drilling holes in the working face (see Fig. 5), and the coal seam, roadway, sandy mudstone and fine sandstone are constructed in the model (Fig. 6).

### Construction of numerical simulation of bolts (cable bolts)

**Figure 7:**
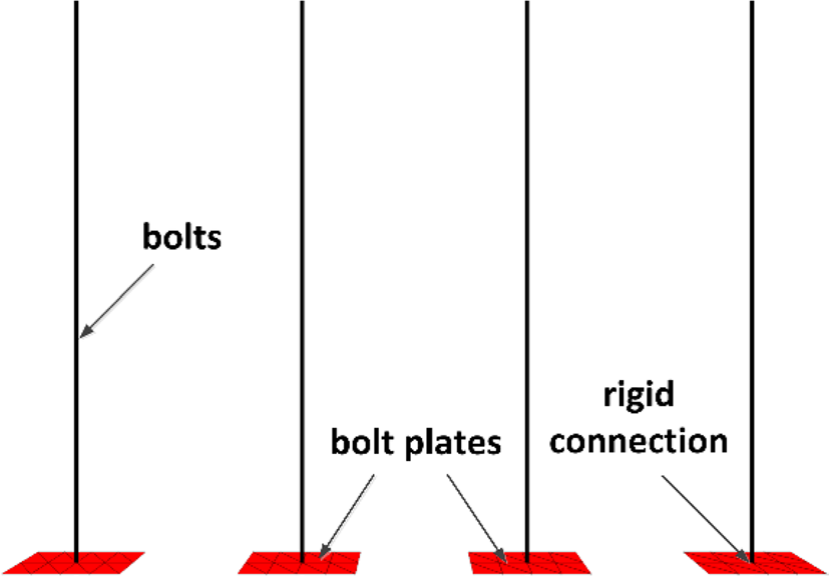
Numerical simulation model of bolts.

**Figure 8:**
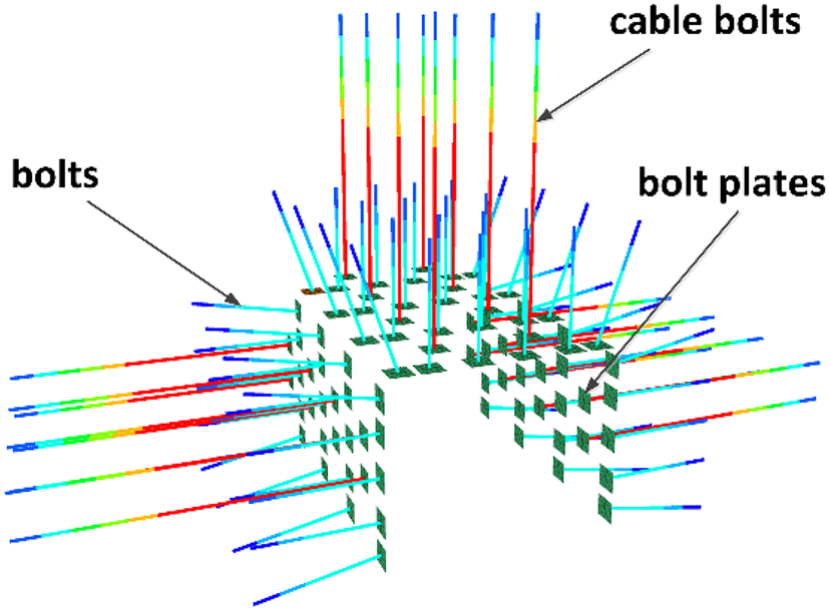
Numerical model of roadway bolts (abchor cables).

In this study, FLAC3D is used for numerical simulation and analysis, and cable structural unit is applied to simulate the bolts and liner structural unit is used to simulate the pallets, and the cable structural unit and liner structural unit are rigidly connected, which can effectively approximate the interaction between the bolts (cable bolts) and the surrounding rock. The numerical model of bolt is shown in Fig. 7, the numerical model of lane bolt (cable bolt) is shown in Fig. 8, the mechanical parameters of bolt (cable bolt) are shown in Table 2, and the mechanical parameters of pallet are shown in Table 3.

**Table 2:** Bolts (cable bolts) physical and mechanical parameters.

Material type	Modulus of elasticity (GPa)	Tensile yield strength (MPa)	Cross-sectional area (m^2^)	Perimeter of grout injection (m)	Preload force (kN)
Rebar bolt	200	500	0.000380	0.1005	80
FRP bolt	60	500	0.000380	0.1005	80
Cable bolt	195	1860	0.000373	0.1005	300

**Table 3:** Pallet physical and mechanical parameters.

Material type	Modulus of elasticity (GPa)	Poisson's ratio	Thickness (m)	Contact surface normal stiffness (N·m^−3^)	Contact surface shear stiffness (N·m^−3^)
Pallets	200	0.25	0.01	8 × 108	8 × 108

### Analysis of numerical calculation results

#### Bolt (anchor cable) axial force analysis

**Figure 9:**
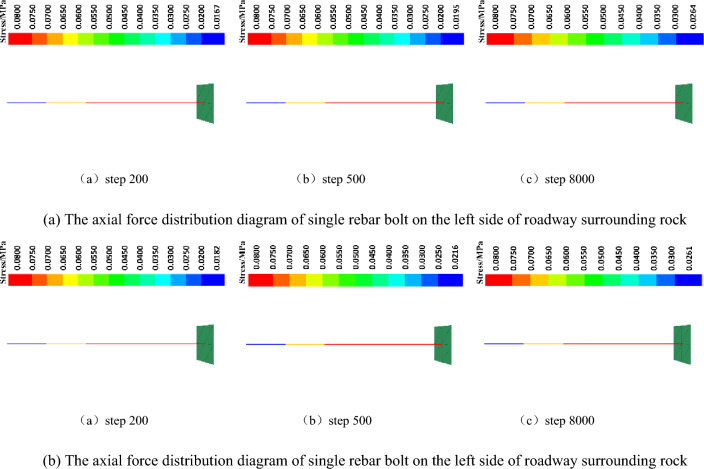
Cables axial force distribution diagram.

The axial force distribution maps of a single rebar bolt and a FRP bolt on the left side of the surrounding rock of the roadway under the two support systems are shown in Fig. 9a and b. The pre-tightening force applied in the free section of the bolt is 80 kN, and the pre-tightening force applied in the free section of the cable bolt is 300 kN. There is no grouting in the free section of the bolt (cable bolt), and the axial force is the applied preload. The axial force change of the tail anchorage section directly reflects the support process of the bolt (cable bolt) to the surrounding rock of the roadway. According to the simulation results, the anchorage section of a rebar bolt has an axial force of 0.0195 MPa before 500 steps of calculation, while the anchorage section of a FRP bolt has an axial force of 0.0216 MPa. The axial force of the anchorage section of a FRP bolt is 11% greater than that of the anchorage section of a rebar bolt.; After 500 steps of calculation, the axial force of the rebar bolt anchorage section is 0.0264 MPa, the axial force of the FRP bolt anchorage section is 0.0261 MPa, and the axial force of the rebar bolt anchorage section is only 1% larger than the axial force of the FRP bolt anchorage section. The difference in axial force between the two is very small. Analysis can be obtained, when the bolt is just in contact with the surrounding rock of the roadway, the elastic modulus of the FRP bolt is small, the resistance to deformation is weak, and the bearing capacity is first; in the bolt and roadway surrounding rock after full contact, the elastic modulus of rebar bolt (200 GPa) is greater than the elastic modulus of FRP bolt (60 GPa), resistance to deformation ability is strong, deformation of the lag over the FRP bolt, until the FRP bolt bearing to a certain degree, then rebar bolts began to occur deformation, the subsequent rebar bolts and FRP bolts to jointly bear the force.

#### Stress analysis of the tunnel surrounding rock

To determine the stress distribution characteristics of the rock around the roadway, it was analyzed the impact of the bolt support, the maximum principal stress, the horizontal displacement, and the vertical displacement.

**Figure 10:**
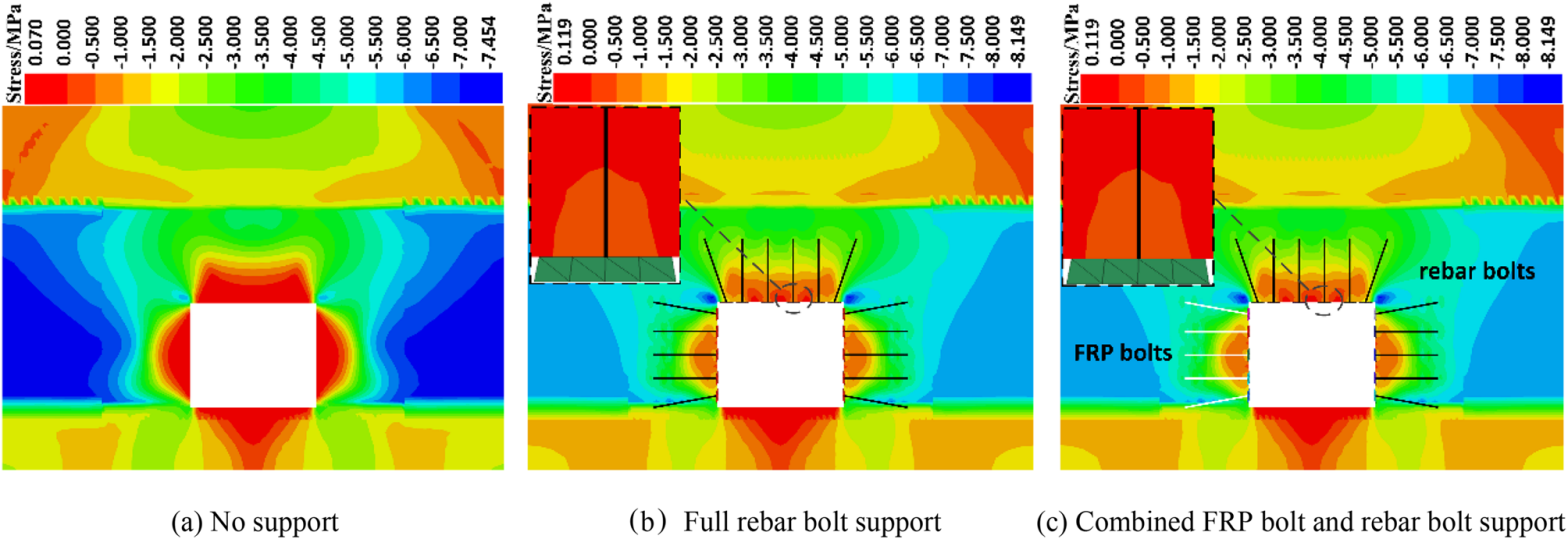
Maximum principal stress.

Figure 10 is the maximum principal stress cloud diagram of roadway surrounding rock under the action of static load without support, full screw steel bolt support, glass steel and screw steel bolt combined support. As shown in Fig. 10a, there is a large range of tensile stress zone in the roof of the roadway when the model is not supported. With the application of bolt support, see Fig. 10b and c, the tensile stress zone of the roof of the roadway gradually decreases, and finally the tensile stress zone of the roof of the roadway is obviously reduced. Under the action of the tray, the roof of the roadway produces compressive stress and transmits to the depth of the surrounding rock of the roadway. The tensile stress on the surface of the roof of the roadway is cut into the morphological characteristics of sporadic occurrence. The simulation results show that the supporting effect of bolt can significantly improve the stress state of roadway roof, reduce or even eliminate the distribution of tensile stress, which fully reflects the improvement effect of support on the stress field of surrounding rock. The surrounding rock of the roadway is mainly dominated by compressive stress. Under the support condition, the stress concentration degree of the four corners of the surrounding rock of the roadway is higher than that without support, and the distribution range is larger. Compared with the rebar bolt support, the combined support of FRP bolt and rebar bolt has no obvious effect on the reduction of the tensile stress range of the two sides and roof of the surrounding rock of the roadway. Under the combined support condition of rebar bolt and FRP bolt, the stress concentration degree on the side of FRP bolt support is less than that on the side of rebar bolt support, but the difference is small.

#### Analysis of roadway surrounding rock displacement

The horizontal displacement and vertical displacement of roadway surrounding rock under different supporting conditions are analyzed, and the displacement distribution characteristics of roadway surrounding rock are obtained.

**Figure 11:**
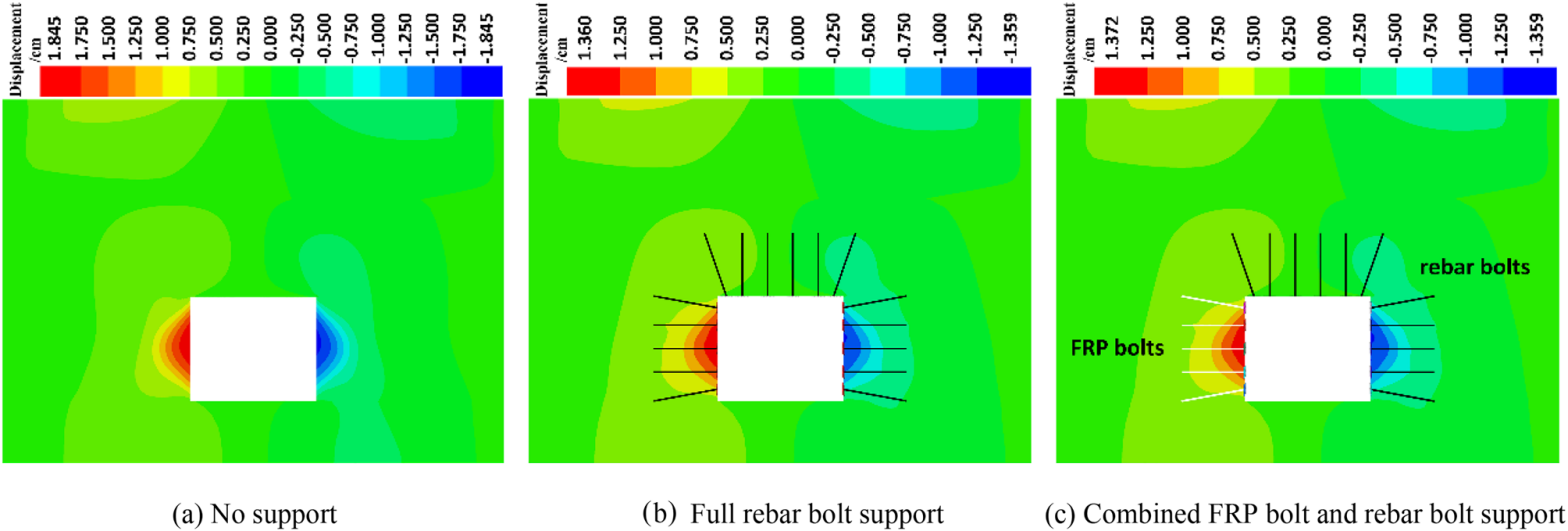
Horizontal displacement.

**Figure 12:**
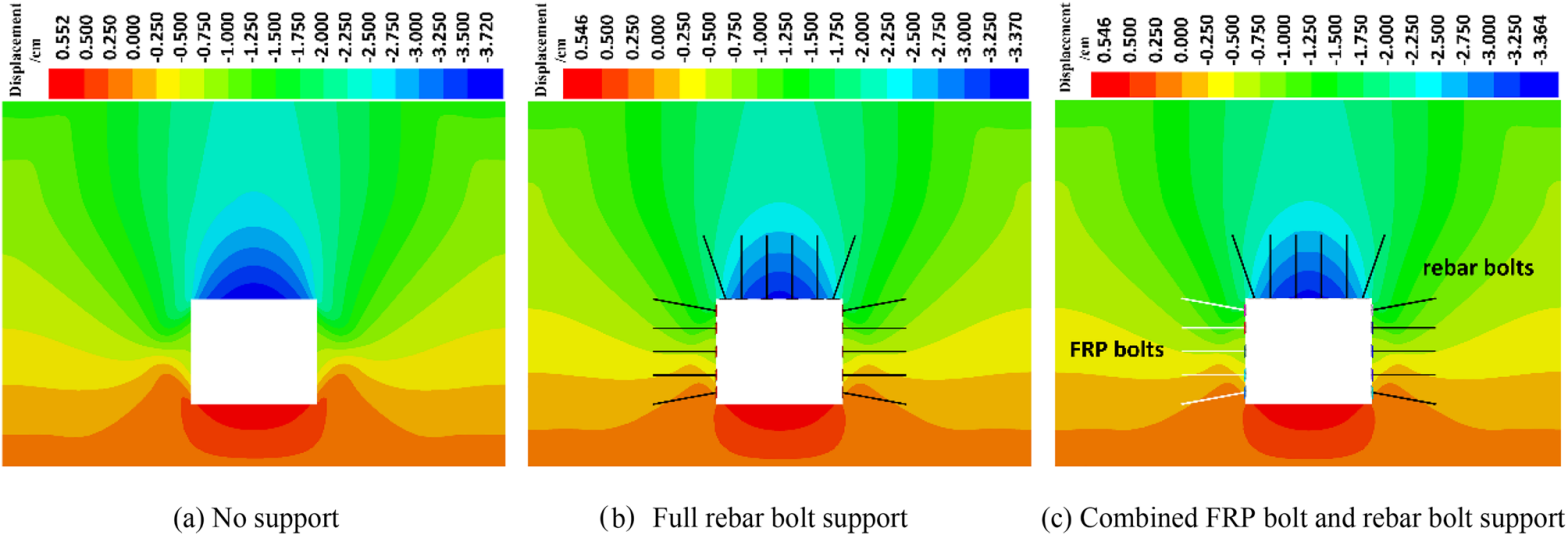
Vertical displacement.

Figures 11 and 12 show the horizontal and vertical displacements of the tunnel rock under static load without support, full rebar bolt support, and combined FRP and rebar bolt support. Compared with the unsupported condition, under the full rebar bolt support and the combined FRP and rebar bolt support, the amount of the two sides of surrounding rock and the amount of roof subsidence are significantly reduced, and the amount of the two sides of surrounding rock and the amount of roof subsidence displacement of the combined FRP and rebar bolt support are approximately the same compared with that of the full rebar bolt support condition. Therefore, the role of full rebar bolt support and FRP and rebar bolt combination support is similar.

**Figure 13:**
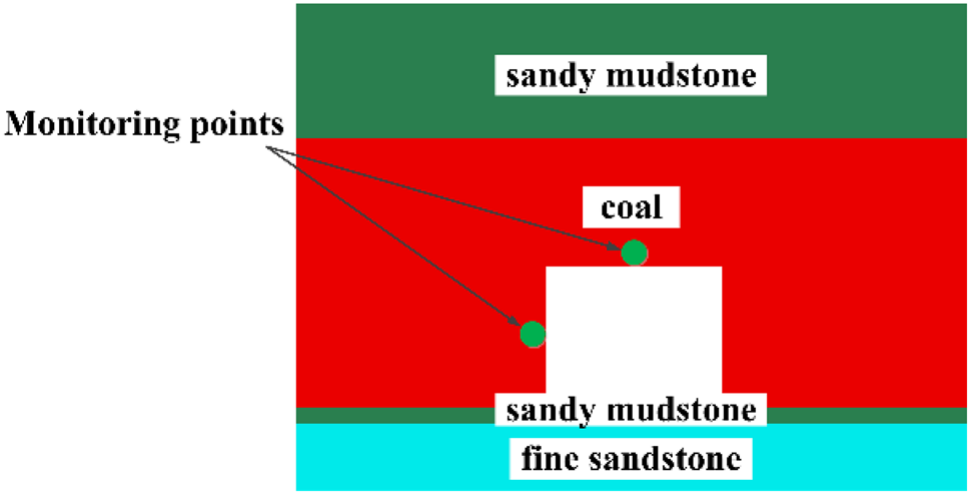
Monitoring point layout diagram.

**Figure 14:**
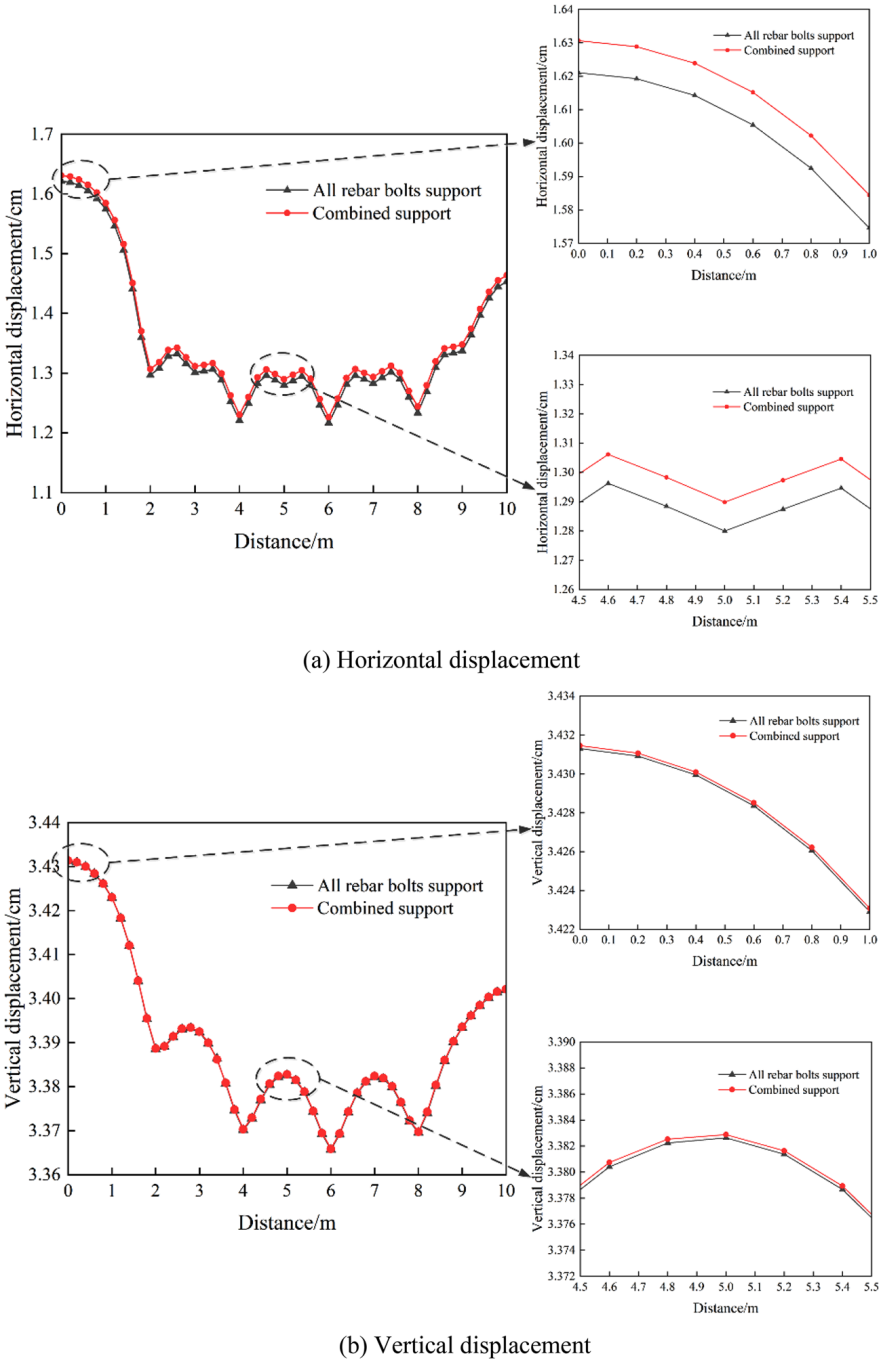
Curve of the displacement of surrounding rock in the roadway.

Figure 14 shows the horizontal displacement of the left side of the roadway perimeter rock and the vertical displacement curve of the roof plate obtained by arranging the displacement monitoring points (see Fig. 13, with the spacing of 0.2 m) and selecting the representative boundary and middle position under the two support conditions of full rebar bolt support and combined support of FRP and rebar bolt. It can be analyzed from the figure, due to the two support conditions only the left side of the roadway support there is a difference, so the left side of the roadway under the two support conditions of the horizontal displacement comparison trend is more obvious than the change trend of the roof plate of the roadway. In addition, the large difference in the monitored displacements between the two ends in Fig. 14a and b is due to the fact that the bolts (cable bolts) are arranged using the fish loop function, which leads to the difference in the unsupported area left at the front and rear ends of the model, resulting in a slight difference in the displacement data between the two ends. The simulation results show that compared with the full rebar bolt support, the maximum roof subsidence is the same with the combination of FRP and rebar bolts, but the maximum horizontal displacement of the left side of the roadway perimeter rock on the FRP bolt support side is 0.44% more than that on the rebar bolt support side. Compared with the non-bolt support, the roof subsidence displacement and the horizontal displacement of the left side of the roadway are effectively controlled. At the same time, the comparative analysis of the full rebar bolt support and the combined support of FRP bolt and rebar bolt shows that under the condition of full rebar bolt support, the control of the roof subsidence displacement and the horizontal displacement of the left side of the roadway is better than the combined support, but the difference is very small. Therefore, it is better to control the deformation of surrounding rock by using the full rebar bolt, but as long as the FRP bolt can give full play to its role, the combined support of FRP and rebar bolt can also control the deformation of surrounding rock, which can meet the needs of the project.

#### Analysis of the distribution of the plastic zone of the roadway enclosure

**Figure 15:**
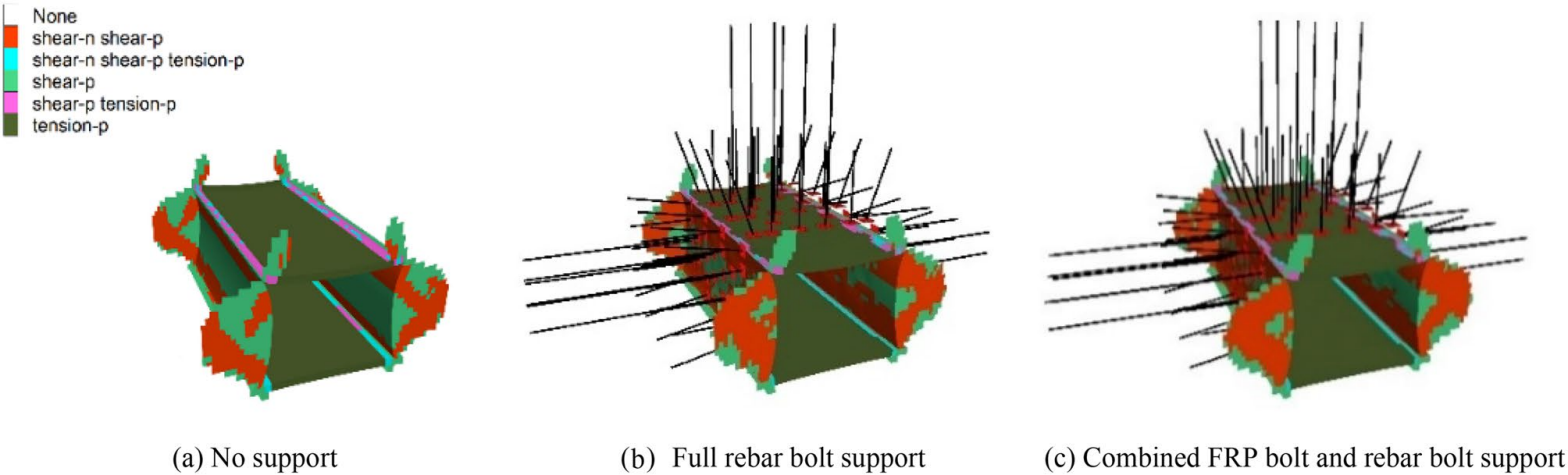
Distribution map of plastic zone of surrounding rock in roadway.

**Figure 16:**
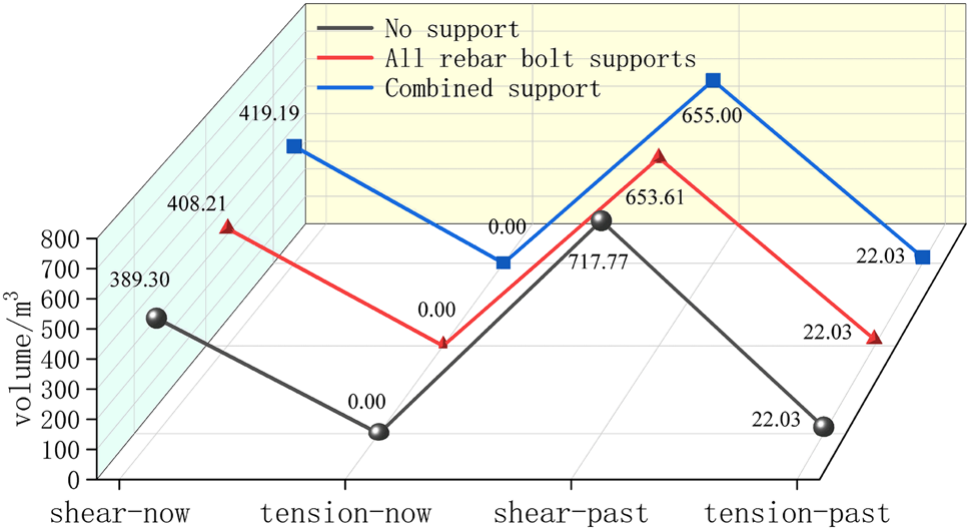
Damage distribution map of surrounding rock plastic zone of roadway.

As shown in Fig. 15, as the stress concentration area shifts from the surface of the roadway surrounding rock to the deep part of the surrounding rock, the plastic zone of the roadway surrounding rock expands, and the strength of the roadway surrounding rock gradually weakens, eventually leading to deformation and failure of the roadway. As shown in Fig. 16, under the three conditions of no support, full rebar bolt support, and combined FRP bolt and rebar bolt support, the volume of the unit in shear damage (shear-now) is 389.30 m^3^, 408.21 m^3^ and 419.19 m^3^, respectively, and the volume of the unit in shear damage (shear-past) is 717.77 m^3^, 653.61 m^3^ and 655.00 m^3^, respectively, and the volume of the unit in tensile damage (tension-past) is 0 m^3^. It is evident that the most common sort of damage to this kind of pavement is shear damage.

In the unsupported condition, the volume of plastic zone is 739.8067 m^3^, accounting for 25.51% of the total volume of 2900 m^3^; in the rebar anchor support condition, the volume of plastic zone is 675.6427 m^3^, accounting for 23.30% of the total volume of 2900 m^3^; and in the combined support condition of fiberglass reinforced plastic and rebar anchor, the volume of plastic zone is 677.0337 m^3^, accounting for 23.35% of the total volume of 2900 m^3^. The volume of the plastic zone in the state of combined support of FRP bolts and rebar bolts was 0.05% larger than that in the state of full rebar bolt support. By comparing the distribution characteristics of the plastic zone of the roadway peripheral rock in the three cases of no support, full rebar bolt support and combined support of FRP and rebar bolt, it can be obtained that: (1) the damage range of the plastic zone in the state of support is smaller than that in the state of no support, and the damage range of the plastic zone of the roadway peripheral rock in the state of anchor support is reduced significantly compared with that in the state of no support, which shows that the bolt support can increase the strength of the roadway peripheral rock and improve the roadway peripheral rock plasticity effectively. (2) The two sides of the surrounding rock of the roadway mainly undergo shear failure. At the beginning, the roof and floor of the roadway mainly undergo tensile failure. After adding bolt support, shear failure occurs. (3) On the side of FRP bolt support, the range of shear failure on the left side of roadway surrounding rock is slightly larger than that on the side of rebar bolt support. The results show that although bolt support can improve the strength of roadway surrounding rock and effectively improve the plastic state of roadway surrounding rock, the support strength of FRP bolt is slightly lower than that of rebar bolt.

## Discussion

In fact, the combined support of rebar and FRP bolts has been maturely applied in many kinds of mines, but the systematic research on the stress, displacement, plastic zone volume and damage type of the roadway surrounding rock under the conditions of combined support of rebar and FRP bolts, as well as the specific axial force distribution characteristics and the support process of the two kinds of bolts combined support are relatively few, so we carried out the relevant research by means of numerical simulation. However, due to some reasons of the coal mine itself, this project has not been implemented yet, and there is no on-site application, and this project is in the stage of project and demonstration. Therefore, there is no way to carry out on-site field monitoring, but we would like to demonstrate the feasibility of the combination of rebar bolt and FRP bolt support through some existing on-site monitoring results.

For many years, the mining roadway and transportation roadway of Jinshan Mine have been supported by rebar bolts. In the process of working face retreating, the roof of the approach is supported by rebar bolts. It is not easy to clear the broken bolts in the caving ore when mining the working face, and it is easy to scratch the belt and reduce the transportation efficiency when transporting. Ye et al.^7^ in order to explore the supporting effect of FRP bolts in the direct roof roadway of weak layer, taking Xiaojiahe Phosphate Mine in Hubei Province as the engineering background, compared with the rebar bolt support, the roadway convergence monitoring test of two kinds of bolt support was carried out. The results show that the deformation law of the roof of the roadway supported by FRP bolt support and rebar bolt support is consistent, and both enter a stable period around the 18th day after monitoring. Zou et al.^23^ carried out the field test of FRP bolt support in Yuhua Temple by taking the sublevel mining approach in view of the defects of the existing screw rebar bolt support in the surrounding rock of Jinshandian Iron Mine, and carried out long-term continuous monitoring and analysis of the roadway deformation displacement and roadway convergence rate of 12 deformation monitoring sections of 4 roadways in the field. The results show that the field support test and convergence deformation trend of FRP bolt and rebar bolt are the same, and the convergence deformation rate is basically the same, indicating that the support effect of the two is the same. In terms of controlling roadway deformation, FRP bolt meets the needs of support. Zhang et al.^24^ in Daye Iron Mine, aiming at the problem that the rebar bolt in the ore-rock contact zone and the powder ore zone of Daye Iron Mine is prone to failure due to corrosion, such as the increase of the aperture, the end force and the failure of the orifice. In order to verify the feasibility of replacing the rebar bolt with the FRP bolt in the powder ore zone and the ore-rock contact zone of Daye Iron Mine, the observation and monitoring of the roadway in the Longdong mining area of Daye Iron Mine were carried out for 6 months. From the perspective of the overall support effect, after the FRP bolt support, to the end of the roadway mining in the FRP bolt support section, no large-scale collapse occurred in the roadway.

It is hoped that our theoretical analysis and numerical simulation results are expected to provide useful feedback for the improvement of the combined support work of rebar bolts and FRP bolts in roadways.

## Conclusion

1. Under the combined support condition of rebar bolt and FRP bolt, in the early stage of the interaction between the bolt and the surrounding rock of the roadway, the elastic modulus of FRP bolt is small, the resistance to deformation is weak, and the bearing capacity is first ; in the late stage of the interaction between the bolt and the surrounding rock of the roadway, the elastic modulus of the rebar bolt is greater than the elastic modulus of the FRP bolt, and the deformation resistance is stronger, and the deformation lags behind the FRP bolt. When the FRP bolt is loaded to a certain extent, the rebar bolt begins to deform, and the subsequent rebar bolt and the FRP bolt share the bearing capacity.
2. Under the condition of rebar bolt and FRP bolt combined support, the stress concentration degree on the side of FRP bolt support is less than that on the side of rebar bolt support, and the horizontal displacement of the left side of the roadway on the side of FRP bolt support is 0.44% larger than that of the left side of the roadway supported by rebar bolt. In addition, the shear failure range of the left side of the surrounding rock of the roadway on the side of the FRP bolt support is slightly larger than that on the side of the rebar bolt support, and the plastic zone volume under the combined support of FRP bolt and rebar bolt is 0.05% larger than that under the rebar bolt support. The results show that although bolt support can improve the strength of roadway surrounding rock and effectively improve the plastic state of roadway surrounding rock, the support strength of FRP bolt is slightly lower than that of rebar bolt.
3. The commonness and difference characteristics of the horizontal displacement of the roadway mining side, the subsidence of the roadway roof, and the plastic zone of the surrounding rock of the roadway under the conditions of the two support schemes are compared and analyzed. It is considered that the FRP bolt is used to replace the rebar bolt in the mining side of the surrounding rock of the roadway. If the FRP bolt is not damaged, the FRP bolt and rebar bolt combined support can maintain the stability of the surrounding rock of the roadway.

## Data Availability

The datasets used and/or analysed during the current study available from the corresponding author on reasonable request.
